# Impacts of G x E x M on Nitrogen Use Efficiency in Wheat and Future Prospects

**DOI:** 10.3389/fpls.2020.01157

**Published:** 2020-07-29

**Authors:** Malcolm John Hawkesford, Andrew B. Riche

**Affiliations:** Plant Sciences Department, Rothamsted Research, Harpenden, United Kingdom

**Keywords:** wheat, G x E x M, nitrogen use efficiency, yield, long-term experiments, phenotyping

## Abstract

Globally it has been estimated that only one third of applied N is recovered in the harvested component of grain crops. This represents an incredible waste of resource and the overuse has detrimental environmental and economic consequences. There is substantial variation in nutrient use efficiency (NUE) from region to region, between crops and in different cropping systems. As a consequence, both local and crop specific solutions will be required for NUE improvement at local as well as at national and international levels. Strategies to improve NUE will involve improvements to germplasm and optimized agronomy adapted to climate and location. Essential to effective solutions will be an understanding of genetics (G), environment (E), and management (M) and their interactions (G x E x M). Implementing appropriate solutions will require agronomic management, attention to environmental factors and improved varieties, optimized for current and future climate scenarios. As NUE is a complex trait with many contributing processes, identifying the correct trait for improvement is not trivial. Key processes include nitrogen capture (uptake efficiency), utilization efficiency (closely related to yield), partitioning (harvest index: biochemical and organ-specific) and trade-offs between yield and quality aspects (grain nitrogen content), as well as interactions with capture and utilization of other nutrients. A long-term experiment, the Broadbalk experiment at Rothamsted, highlights many factors influencing yield and nitrogen utilization in wheat over the last 175 years, particularly management and yearly variation. A more recent series of trials conducted over the past 16 years has focused on separating the key physiological sub-traits of NUE, highlighting both genetic and seasonal variation. This perspective describes these two contrasting studies which indicate G x E x M interactions involved in nitrogen utilization and summarizes prospects for the future including the utilization of high throughput phenotyping technology.

## Introduction

**Figure 1:**
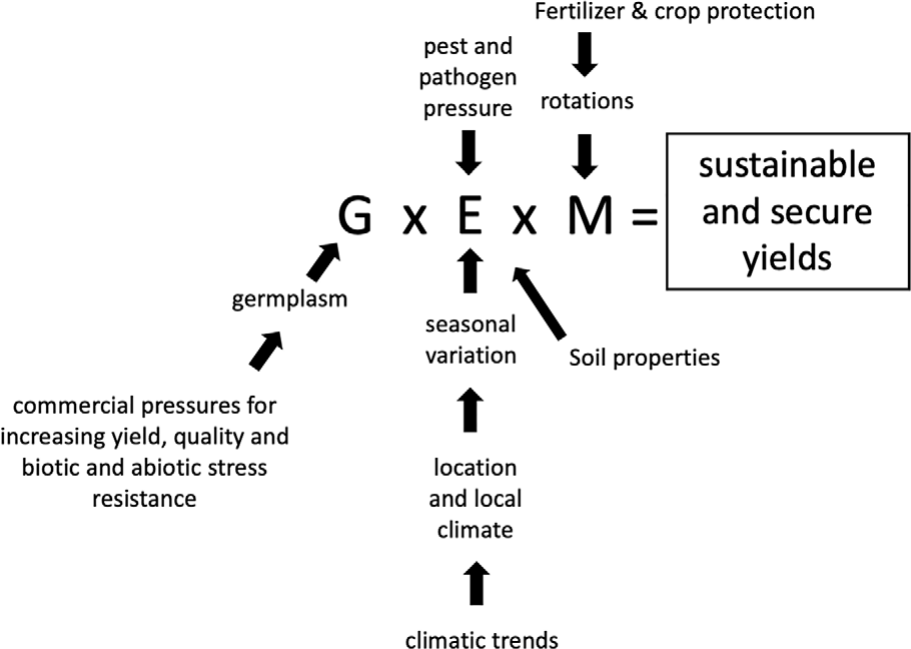
G x E x M is influenced by multiple factors but ultimately determines sustainable and secure crop yields.

Evaluation of crop performance needs to consider genetic variation (G), environmental conditions such as climate (including annual variability) and location (E), together with farm agronomic management (M). It is the combination of these parameters and their interactions (G x E x M) which will determine sustainable and secure crop yields. Each parameter is composed of, or determined by, specific factors as indicted in **Figure 1**. Breeders continually seek to improve performance in terms of yield potential, improved quality and resistance to stress, both biotic and abiotic, by introducing new, mostly higher yielding varieties to compete and succeed in a commercial environment. In addition, management practices evolve, making better use of improved technology and knowledge as well as new varieties. Principal challenges are tackling pest and disease resistances whilst having to cope with a reducing range of pesticides, due to legislation banning some chemistry and requirements for increased environmental protection, thereby setting limits on chemical use. At the same time, reducing farmer incomes in many regions, with costs increasing disproportionately to output prices, alters the economics of wheat production, with a greater yield response required to cover input costs such as fertilizers.

Globally, wheat yields have steadily increased over time as a result of genetic improvement and better agronomy. Regional wheat yields also differ considerably across the globe and locally vary on an annual basis, primarily depending upon fluctuating climatic conditions, but also as a result of pest and pathogen pressures. Plant breeding seeks to increase yield potentials and produce more resilient germplasm able to resist these abiotic and biotic stresses. Agronomic practice also seeks to negate or at least moderate the influence of these factors and enable farmers to approach yield potentials for any particular crop and environment. In recent years the trend of increasing annual yields of some crops may have plateaued in some environments, for example wheat in northwest Europe (Grassini et al., 2013). Whilst the reasons for this are multifold, overcoming such yield limitations will require both genetic and agronomic approaches and will need to account for the influence of climate change. Whilst major agronomic developments have had large impacts on yield in the past, in more recent times genetic improvement has been increasingly important for crops such as wheat (Mackay et al., 2011). Anticipating future climatic impacts on yield will require the understanding of genetic, management and environmental effects, and importantly their interactions.

The need to improve yields with efficient fertilizer use has led to a number of G x E studies on breeding trends for wheat crop improvement and NUE traits specifically. For example studies have focused on partitioning of N between tissues (Foulkes et al., 1998; Gaju et al., 2011; Barraclough et al., 2014), variation in photosynthesis and impacts on yield potential (Gorny et al., 2006; Gaju et al., 2016), and kinetics of senescence which influence both yield potential and nitrogen remobilization and partitioning (Gaju et al., 2011). Variation in NUE traits has also been shown in an analysis of historical wheats and substantially explained by phenological and morphological traits such as flowering time and height (Guttieri et al., 2017). Other germplasm studies have focused on traits relating to abiotic stresses such as water use efficiency, simultaneously linking environmental stress to grain N traits (Sadras and Lawson, 2013). Quality traits, including those associated with N content, may be genetically controlled, or may be strongly influenced by environment, as shown by an analysis of data on 316 German varieties (Laidig et al., 2017).

A widespread management strategy which has a major impact on crop performance and has implications for NUE is crop rotation. Individual crops should not be considered in isolation and NUE should be evaluated as part of the whole cropping system when considering economic or environmental impact. For example, one modeling study highlighting the importance of looking at the cropping system and not just individual crops, clearly identifies that NUE of one crop will impact on succeeding crops (Dresboll and Thorup-Kristensen, 2014).

Further management practices critical in influencing NUE will be the variable utilization of N-fertilizer depending on timings, amounts and chemical formulation. For example, coated urea for controlled release of N, more effective timing of applications, and appropriate dose rates, taking into account soil N supply, to optimize yield and quality whilst minimizing losses and avoiding contravening legislation. A recent overview outlining strategies for reducing crop N requirements highlights the importance of taking a holistic view combining elements of improved germplasm and agronomy (Swarbreck et al., 2019).

## Defining Nitrogen Use Efficiency

To consider the impacts of G x E x M, as individual factors as well as in combination, on NUE, it is necessary to define NUE and consider how the key constituent components may be affected individually and as a whole. NUE may be considered as the efficiency of nitrogen recovery from applied fertilizer, or from the N available to the crop, and this gives rise to the 33% efficiency of crop recovery (Raun and Johnson, 1999; Zhang et al., 2015). Alternatively, it is often considered as a productivity index and defined as the yield produced per unit of available N (Moll et al., 1982; Barraclough et al., 2010). Another distinct definition of NUE is to consider nitrogen responsiveness in combination with dose response curves to identify economic N-optima (Swarbreck et al., 2019).

**Figure 2:**
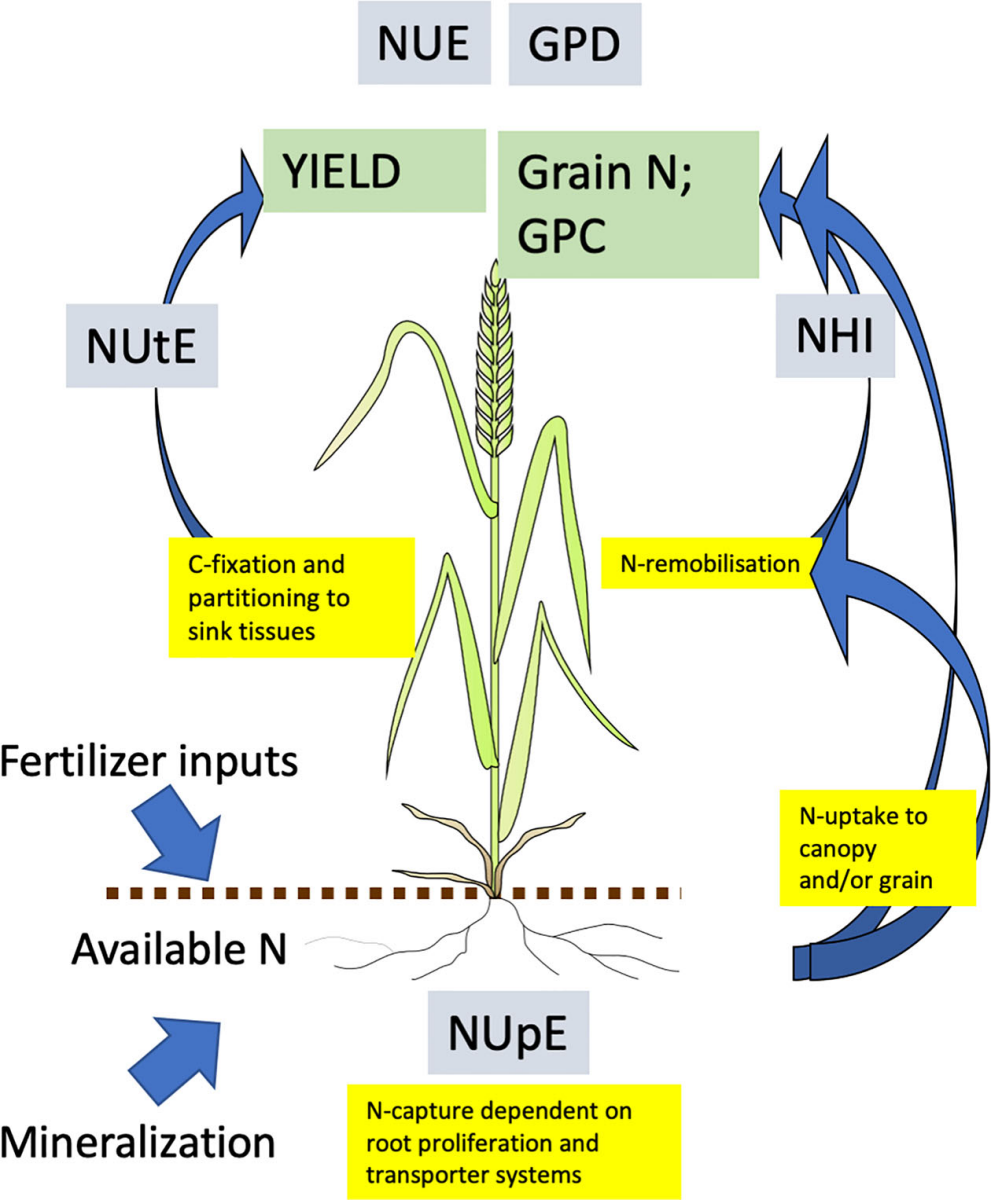
Processes contributing to and determining NUE in wheat. Measures of nitrogen use efficiency shown in grey boxes; primary traits in green boxes; physiological process in yellow boxes. All abbreviations are in the text. Arrows indicate movement of N. Adapted from (Hawkesford, 2011).

Whichever definition is used, plant growth and yield require nitrogen, and furthermore are dependent upon multiple physiological processes (**Figure 2**). From this perspective, it is useful to use the productivity index and the component traits of this index (Barraclough et al., 2010) to consider impacts on G x E x M. NUE may be considered the top level trait and for wheat is the yield of grain produced per unit of N available to the crop; it is expressed as kg yield per kg of available N; it is also the product of the two second level traits, N uptake efficiency (NUpE) and N utilization efficiency (NUtE). NUpE, or sometimes biomass NUpE (BioNUpE) is the ratio of N taken up by the crop compared to what is available from the soil and applied fertilizer, and is expressed as kg N (in the crop) per kg N (available). N in the roots is ignored, but the N in the aerial biomass for wheat is that in the grain and straw combined. NUtE or grain NUtE is the amount of grain produced per unit of N taken up, and is also kg (grain) per kg N. NUE is mathematically the product of NUpE x NUtE. An interaction between N-management and genetic variation of these second level traits was indicated in a two-site and four-year tial of 16 wheat genotypes, in which genetic variability in NUtE rather than NUpE was reported to be of greater significance at low N inputs (Gaju et al., 2011).

Other useful measures of efficiency of N use include the nitrogen harvest index (NHI), which is the fraction of total N taken up by the crop which is partitioned to the grain, and is a refinement of harvest index (HI) which descibes partioning of dry matter alone. However, NHI is independent of yield and uptake efficiency, and a low yielding crop may have a high NHI, but leave substantial unrecovered N in the soil. High grain protein concentration (GPC) is required for end-uses such as flour for breadmaking, however, it is difficult to increase GPC without decreasing yield due to the negative relationship between the two, as high yield usually reflects high carbohydrate content which in turn dilutes N concentration. The desired trait, to increase GPC without reducing yield, can be defined as grain protein deviation (GPD), the deviation from the negative linear relationship between yield and N concentration, and reflects an ability to acquire more N in the grain for a given yield (Bogard et al., 2010; Mosleth et al., 2015). There is some uncertainty of the physiological basis of GPD but it may be related to phenology and post anthesis N uptake (Bogard et al., 2010; Bogard et al., 2011). Both GPC and GPD may be overcome agronomically with higher N-inputs, particularly later in the season when yield has been largely determined, however this inevitably leads to low NUE. Future research may develop techniques for making bread from low protein wheats, which would be a major breakthrough for increasing NUE whilst maintaining end-use suitability, although the reduced protein content may be detrimental for a healthy diet.

Each of these NUE parameters are complex traits involving many underpinning physiological and biochemical reactions and pathways. Genetic studies indicate the multigenic and heritable nature of the major traits and the underpinning processes. However, unravelling the traits and breeding for improved NUE is complex. Genetic variation in many traits is apparent in modern germplasm and to an even greater extent in historic material, landraces and wild relatives, and could be the basis for germplasm selection. Yield is commonly the major commercial target for selection, usually at a constant N input, hence selection for NUE and NUtE is consequentially also selected for. Differentiation between NUpE and NUtE is not made consciously, however higher yielding, high protein genotypes (and hence high GPD types) will have high NUpE also. Efforts have been made to consider management protocols (M) by including selection at different N-inputs (Ortiz-Monasterio et al., 1997), however a common assumption is that ranking of variety performance is independent of N-availability. Growers will also be looking to maximize profitability, which may be different from maximizing NUE, particular if they are aiming for a quality market.

Arguably the greatest genetic improvement (G) has been the introduction of short straw (dwarf) varieties, and as a consequence, HI and NHI have increased. The immediate effect is that biomass allocation to the grain is favored, maximizing grain yield at the expense of straw biomass. Another direct consequence of utilizing dwarf varieties is that the reduced stature facilitates resistance to lodging, a problem particularly encountered at high levels of N fertilization, particularly when combined with conditions of high wind and rainfall. The ability to exploit higher N-rates has led to a management strategy (M) of increased N-inputs, which promotes greater yields but at lower efficiency. Higher N fertilization can increase disease and weed pressure, requiring additional agrochemical inputs.

The chief environmental factors (E) consider location (and soil type) and local biotic and abiotic stresses. In the latter case heat and drought are the most major impactors limiting yield and decreasing N requirements (Halford, 2009). Long term environmental factors will be global temperature and CO_2_ trends, which are affecting yield potentials and can be predicted to have substantial influences in the future by altering timing of phenology or favoring photosynthetic processes determining NUtE (Semenov and Shewry, 2011; Asseng et al., 2019). Climatic changes will also impact on rainfall patterns and influence nutrient availability, requiring optimized root related traits favoring high NUpE.

These genetic, environmental and management interactions in nitrogen fertilizer use and expression of crop NUE traits are amongst the clearest examples of the importance of G x E x M. Fertilizer use underpins crop performance and the interactions between these factors is complex but vital for efficiency. An exemplar dataset is described in the next section.

## Broadbalk as a Historic Exemplar G x E x M Experiment

**Figure 3:**
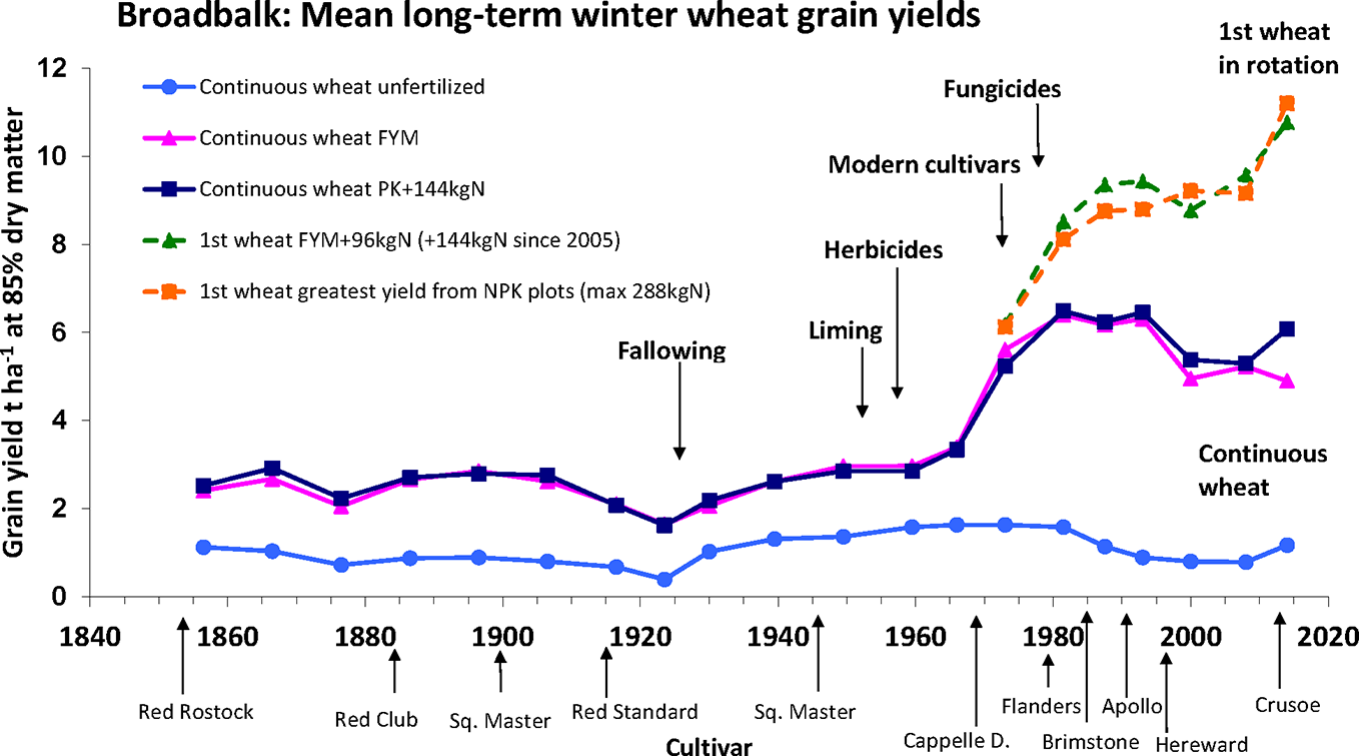
Yield data for selected treatment on the Broadbalk long term experiment at Rothamsted from 1852 to 2016. Taken from ‘Broadbalk mean long-term winter wheat grain yields’ (Rothamsted-Research, 2017) and used with permission under a creative Commons Attribution 4.0 International Licence.

The Broadbalk long term experiment, which was initiated in 1843 at Rothamsted Experimental Station, in the United Kingdom, is the world’s oldest continuously running agricultural experiment (Johnston and Poulton, 2018). Originally conceived as an investigation into nutritional requirements for wheat growth, the continuous records, varied inputs and the modifications to crop management that have been put in place over the course of the experiment, coupled with a range of varieties grown, each for periods of several years, contribute to making this an exemplar long term G x E x M experiment. The experiment has been recently fully described and datasets are available electronically on request (Johnston and Poulton, 2018; Perryman et al., 2018). Yields from 1852 to 2016 for selected treatments are shown in **Figure 3**. Long term trends in yield responses are clearly seen, and whilst year to year variation is observed, this is not apparent in the figure as the data is presented as multi-year means. Due to multiple factors included over time, these datasets represent a valuable resource for investigating G x E x M, and are all available on request (Perryman et al., 2018).

Progress in wheat breeding (G) is demonstrated as, since the start of the experiment, the variety planted has been changed periodically, and has been usually a variety commonly in use commercially at the time. The major change was the adoption of shorter strawed varieties from Capelle Desprez onwards in 1968. While all varieties have good yield potential, the modern shorter varieties are better adapted for the higher N-inputs, as they are less likely to lodge. The development of plant growth regulators has also enhanced the effect of shorter varieties and further reduced the occurrence of lodging.

The dataset is ideal for examining long term trends due to climatic factors (E) over a considerable period of time, however analysis is complicated by changing agronomic practice, which again, has followed typical commercial practice and, along with improving genetics and changing varieties, has contributed to increasing yields. One recent study focused on datasets from 1968 to 2016 to minimize some the impact of the changing agronomic practice but still enable longer term trends to be evaluated. This study highlighted the strong climatic influence on year to year variability of yield and N-responses in wheat, and also barley in a separate experiment, of particularly temperature and rainfall in specific months (Addy et al., 2020).

Several management interventions (M) are represented in the Broadbalk experiment. Key amongst these are the rotations and specifically the comparison between continuously grown wheat and the first wheat in a 5-year rotation comprising successive wheat crops combined with break crops. A first wheat outperforms the continuous wheat partly due to a lower root disease pressure. Other notable agronomic practices having a positive effect on crop performance are the introduction of herbicides and fungicides. While the former replaced manual weeding or fallowing, the latter became essential with increased canopy disease favored by the greater canopy biomass achieved from the addition of high rates of nitrogen. The key agronomic treatments with respect to NUE are the differential rates of N, with higher N inputs resulting in increased yield, particularly with modern varieties, variations in the patterns of applications (single versus split doses) and forms of applied N (inorganic versus organic sources). The Broadbalk experiment has also been used to investigate, utilizing buried drains, the detrimental effects of N leaching, which occur following excess or inappropriately timed N applications, and will reduce NUpE.

As illustrated above, the Rothamsted “classical” long-term trials are an ideal dataset to examine long term trends due to climatic factors over a considerable period of time, since their inception in 1843. However, for any single year these trials lack the multi-germplasm genetic factor. Therefore many recent trials have sought to introduce genetic variation by working with germplasm panels or multi variety datasets (Foulkes et al., 1998; Gaju et al., 2011; Mackay et al., 2011; Sadras and Lawson, 2013; Guttieri et al., 2017; Laidig et al., 2017). One such study is described below, and early data were reported by Barraclough et al. (2010).

## Recent Wheat Germplasm Study in the UK Focused on NUE

**Figure 4:**
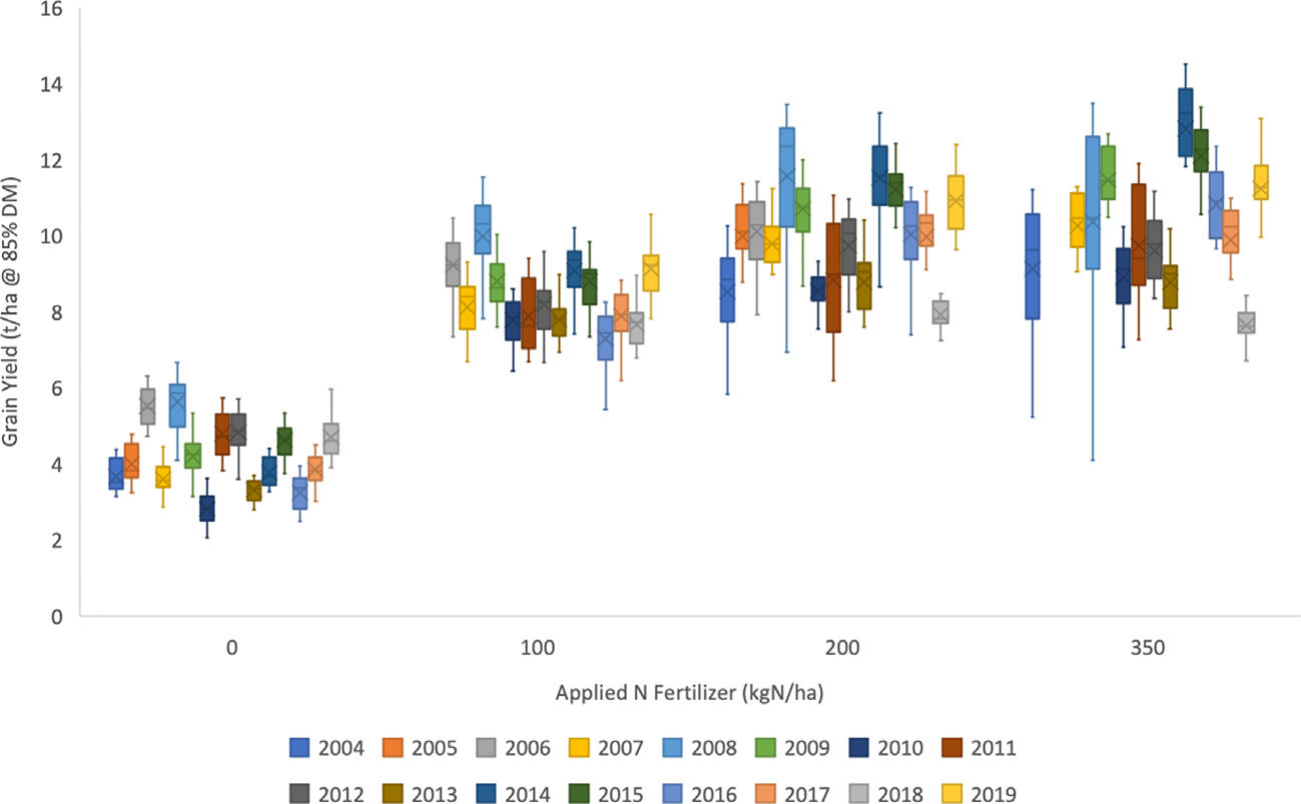
Annual means of 15 wheat varieties (as listed in ) for four rates of applied fertiliser-N (0, 100, 200, and 300 kg N/ha), for the duration of the UK Department for Environment, Food and Rural Affairs (Defra)-funded Wheat Genetic Improvement Network (WGIN) trials from 2004 to 2019). The median and upper and lower quartiles are shown for each year at each level of N. There was no N350 treatment in 2005 and 2006; no N100 in 2004 and 2005; and no N0 in 2019. Data available at http://www.wgin.org.uk and is described in (Barraclough et al., 2010). Maris Widgeon, Paragon and Soissons outliers were excluded from the plot.

**Figure 5:**
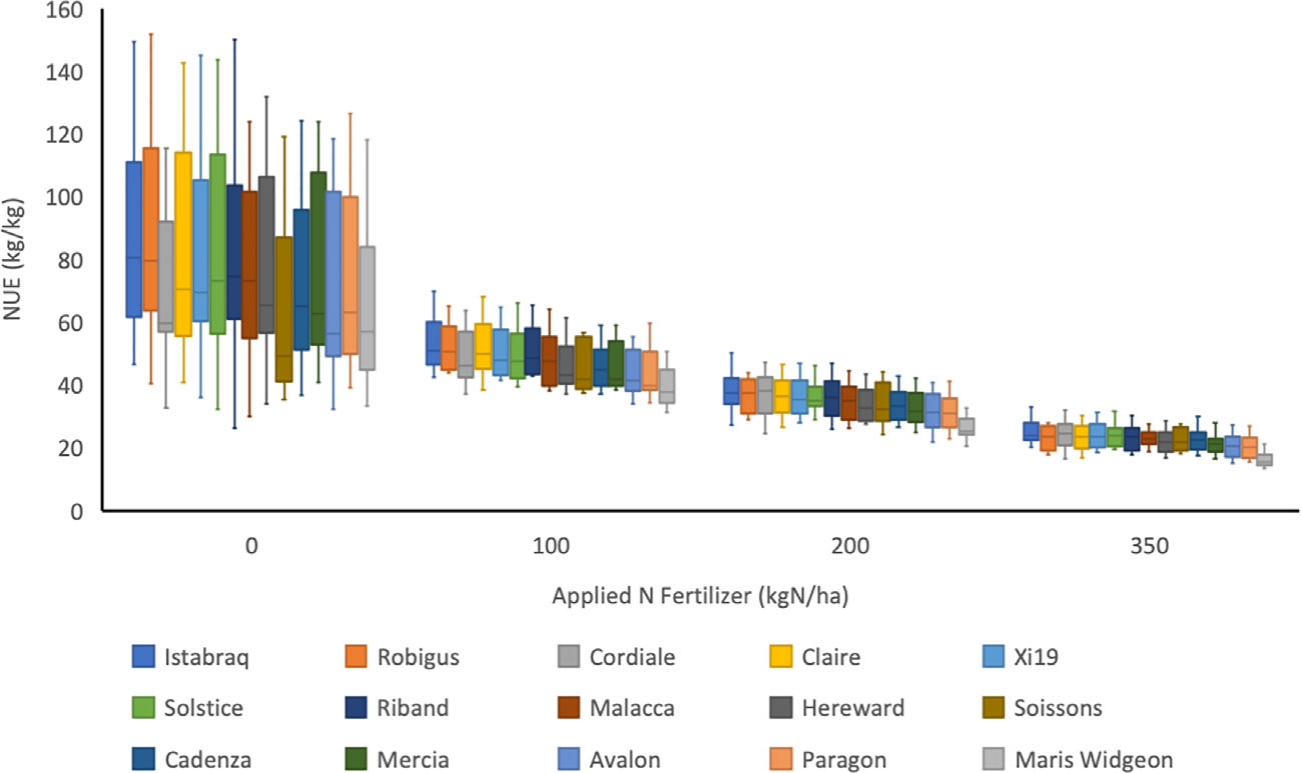
Calculated NUE (kg/kg) for 15 wheat varieties in a UK trial between 2006 and 2017 grown at four levels of nitrogen fertilisation (0, 100, 200 and 350 kg N/ha; N0, N100, N200 and N350, respectively). Soil available N ranged from 25.6 to 115.7. The median and upper and lower quartiles are shown for each cultivar at each level of N. There was no N350 treatment in 2006, and no Soissons data in 2017. The trial was located at Rothamsted Research in the UK and was part of the UK Department for Environment, Food and Rural Affairs (Defra)-funded Wheat Genetic Improvement Network (WGIN) project. Data available at http://www.wgin.org.uk. Malacca and Maris Widgeon outliers were excluded from the plot.

**Figure 6:**
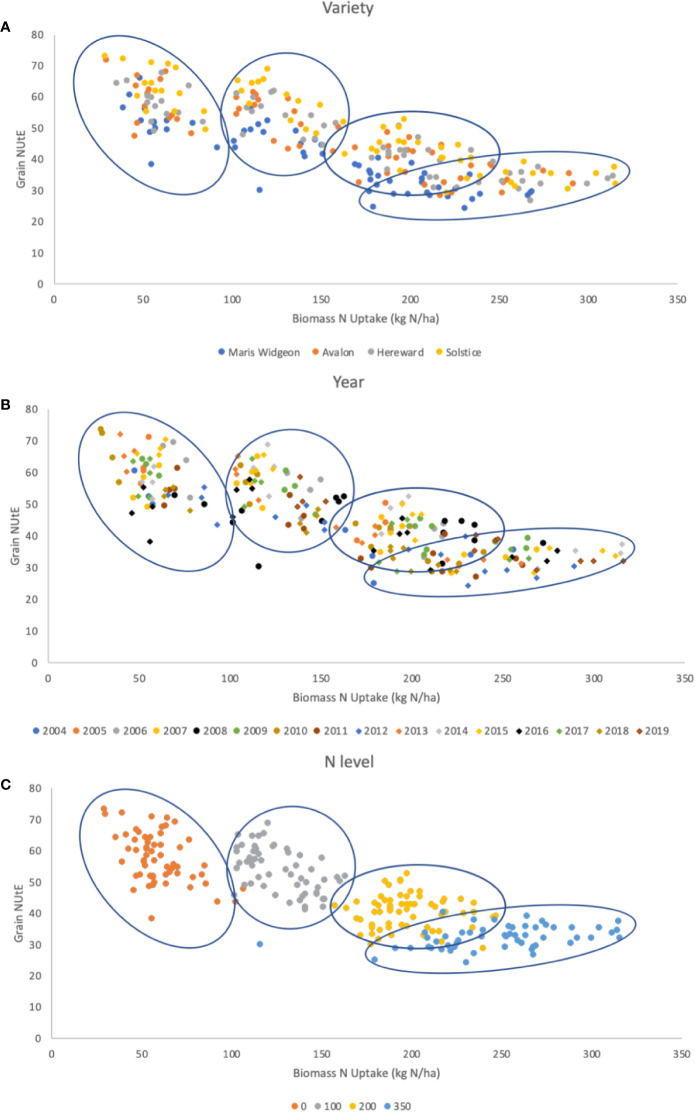
G x E x M for the relationship between grain NUtE and total biomass N uptake. Data are for 4 varieties (G), Maris Wigeon, Avalon, Hereward, and Solstice, for trials harvested from 2004 to 2019 (E), for at 4 different N input rates (M), 0, 100, 200, and 350 kg N/ha. Data points are colored to indicate **(A)** G, **(B)** E, and **(C)** M. The clusters of data points apparent due to the different N-inputs [panel **(C)**] are circled in each of the panes **(A**–**C)**. Slopes, intercepts and R^2^ of data points for **(A)**: overall -0.129, 66.23, 0.666; Maris Widgeon -0.134, 60.374, 0.746; Avalon -0.1322, 67.177, 0.7273; Hereward -0.1243, 66.843, 0.7959; Solstice -0.141, 72.97, 0.8058. Slopes, intercepts and R^2^ of data points for **(C)**: overall -0.129, 66.23, 0.666; N0 -0.2532, 72.613, 0.2454; N100 -0.2156, 80.99, 0.2903; N200 -0.0162, 43.367, 0.0038; N350 0.0354, 23.272, 0.1438. There were no significant regressions based on year **(B)**. Data available at http://www.wgin.org.uk.

The Wheat Genetic Improvement Network (WGIN) germplasm diversity trial is an example of a multi-variety, multi-N treatment series of trials conducted over multiple years. Data from the initial years (2004–2008) of these trials reported variation in yield and N-responses and contributing physiological processes (Barraclough et al., 2010; Barraclough et al., 2014). The trials have continued to the present date and have involved a large panel of modern commercial hexaploid wheats (varieties introduced between 1964 and 2016) and data is summarized here for trials from 2004-2019 (**Figures 4**–**6**). All data are available on the WGIN website (http://www.wgin.org.uk). In most years there were four N rates, from zero to 350 kg N/ha/yr, which represents no input through to excess applied N. All trials were conducted following local commercial agronomic practice, at the Rothamsted Farm in Hertfordshire in the UK. Whilst more than 60 varieties were examined in total, a smaller subset of 15 core varieties have been grown for most years. The mean grain yield trends of this core set for the 4 N rates over the period of the trial are presented in **Figure 4**. A substantial yield increase in response to fertilizer (N100, N200, and N350) compared to no fertilizer (N0) is seen for all years. A modest increase is seen for rates above 100 kg N/ha (N100), however there was little difference between the two higher rates of 200 and 350 kg N/ha, N200 and N350, respectively. Substantial year to year variation was apparent from 2004 to 2019, with some years having notably low yields (2007, 2010, 2016) and other years having notably higher yields (2008, 2009, 2014, 2015, and 2019). It is likely that the year to year variation was principally due to variations in weather patterns in the individual years. These annual variations have a direct impact on crop growth, and also influence management, for example, wet weather in early spring can delay N applications due to the soil being too wet to drive on with the application machinery, and similarly, wet weather in the autumn can delay drilling; both of these may affect yield and consequently NUE. The same yield variation patterns were apparent for all N levels, with little indication of any year by N interaction. Notably there were no major long-term trends apparent.

An indication of genotypic variation in a high-level nitrogen use trait, NUE, and the interaction with the N fertilizer treatments is illustrated in **Figure 5**. NUE as defined as grain yield per unit of available N (fertilizer and mineral soil N) was determined for the same panel of 15 commercial modern wheat varieties whose mean performances are presented in **Figure 4**. In **Figure 5**, variety data is presented as the means over the period 2006–2017, years for which data at all 4 N-rates was available.

Applied N impacts on yield, however this response is non-linear (see **Figure 4**), with the marginal yield increase decreasing as N rates increase, and therefore NUE progressively decreases as N-inputs increase (**Figure 5**). NUE is highest at the lowest N-rate (N0) but at this rate all varieties also have the widest range of values. There is genetic variation apparent in NUE, which reflects the range of yields, with the highest yielding varieties having the highest NUE. The ranking of the varieties at each of the N-rates is almost identical and therefore appears to be independent of the N-rate.

A more detailed analysis of G, E, M and their interactions for 4 varieties within this dataset is presented in **Figure 6**. These 4 varieties are potential milling quality varieties and are representative of the development of UK wheat over a 40 period; Maris Widgeon was introduced in 1964, Avalon 1980, Hereward 1991 and Solstice 2002. Grain NUtE is plotted against total N taken up by the crop at harvest. In each of the 3 panels the data points are highlighted with color schemes to show the distribution of responses based on variety (**Figure 6A**), year of harvest (**Figure 6B**) and N input level (**Figure 6C**), G, E, and M, respectively. The clearest clustering is due to the 4 N-rates as shown in Fig 6C and these clusters are circled in all three panels to aid visual comparisons. Overall, taking data from all N-inputs, there is a negative relationship between NUtE and N taken up. However, within an N rate, NUtE and N-uptake are poorly correlated, indicative that these are quite distinct physiological processes. **Figure 6A** illustrates that different varieties have different NUtE irrespective of the N-rate, indicative of the intrinsic yield potentials of the separate varieties, with Maris Widgeon (the oldest variety in the panel) generally having the lowest and Solstice the highest NUtE at any given N-uptake. There is no evidence that there is any relationship of variety to N-uptake for any given N-rate. Examination of Fig 6B indicates some weak clustering of data points due to year of the trial, reflecting higher or lower yielding years. **Figure 6C** clearly indicates the clustering of data points due to the N-rate. N uptake increases with increasing N-rate. Higher N-availability promotes biomass yield (which will increase NUE), increasing total N-uptake and promotes higher grain N-content (which will decrease NUE) in terms of concentration (data not shown). NUtE is notably higher at the lowest N-rates because of the non-linear relationship between yield and N-rate, as also seen in **Figure 4**. Within any individual N-rate there is no strong correlation evident between NUtE and N taken up, underling the independent nature of these traits as noted above (see **Figure 2**) and additionally reflecting the year to year variability of performance, shown also in **Figure 4**. At the lower N-rates, and particularly at zero (N0), N-uptakes varied widely with little variation in NUtE; this may at least partially due to NUpE reflecting variations in soil N seen between sites used in individual years of the trial.

In summary, within this germplasm panel, the N-rate, as the major management treatment (M), is the dominant factor. Variety (G), differentiating higher and lower yielding types, and year (E) (higher and lower yielding seasons) also have roles in determining yields, N-uptakes and NUtE. Whilst all three factors and their interactions determine yield and NUE, a clear understanding of the interactions of G x E x M will require larger and more detailed datasets.

## Prospects: Research Gaps and Using Automated Phenotyping for High Resolution Data Collection in Field Studies

The Broadbalk experiment and similar trials are extremely useful for examining long term trends in (wheat) crop performance and additionally illustrates importance of variety and management. Trials of diversity panels, such as that described here, enable an examination of the genetic components influencing crop performance, however examining the importance of environment and management become a major undertaking in terms of scale and investment of resources. A major gap in effective G x E x M analysis is in having enough contrasting environments with appropriate germplasm and management ranges. An elegant solution is to conduct meta-analyses, bringing together multiple studies. An example is an analysis of 55 individual studies conducted between 1974 and 2014 in multiple global locations, in which a clear non-linear relationship between yield and N uptake was observed and indications of greater opportunities for improved NUtE at higher yielding sites (de Oliveira Silva et al., 2020).

**Figure 7:**
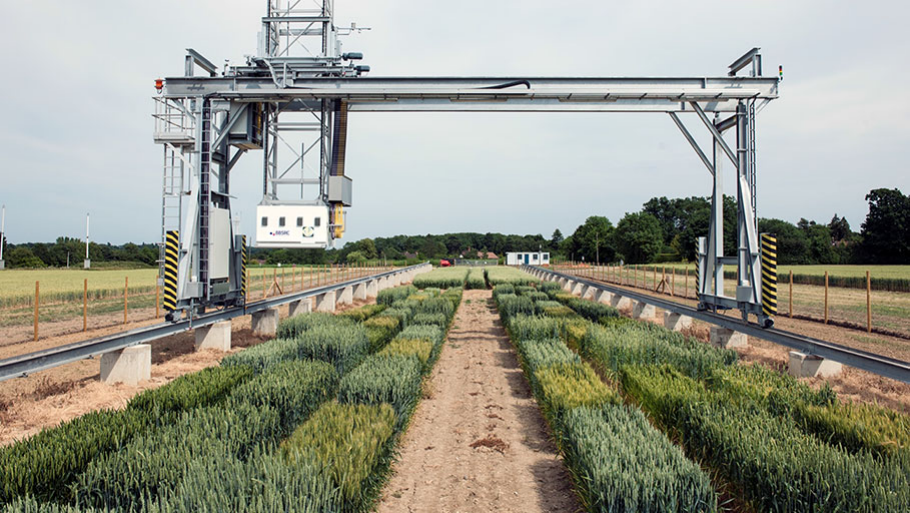
Automated phenotyping technology. A field-located automated phenotyping system at Rothamsted Research in the United Kingdom (Virlet et al., 2017). Multiple sensors are located in a platform which can be positioned in 3 dimensions above a growing crop and can record parameters relating to growth and health.

In addition to the challenges of larger trials conducted at multiple sites and in multiple years, there is an increasing demand for higher resolution data, both spatially and temporally. Solutions to this challenge exploit new technologies for automated and high throughput phenotyping, for example using remote sensing and robotics. An example of an automated robotic system is shown in **Figure 7**. This programmable system contains a range of image-based sensors with specific spectral sensitivity mounted in a positionable-platform which can be used for autonomous collection of high-resolution datasets. Plant growth and health parameters are extracted from the collected images (Sadeghi-Tehran et al., 2017; Virlet et al., 2017; Sadeghi-Tehran et al., 2019). Detailed datasets can reveal hitherto unrecognized information concerning the genetic control of performance revealed at different developmental stages (Lyra et al., 2020). Similar datasets can be obtained from drone-based platforms which are able to cover larger trials at multiple sites, but require greater manual inputs for collection (Holman et al., 2016; Holman et al., 2019).

For most high throughput technologies emphasis has been placed on growth and biomass accumulation, both indicators of final performance and yield. Such data may be derived from height measurements or spectral indices, indicative of canopy cover. In addition, spectral parameters, including the growth indices mentioned above, are measures of chlorophyll content and hence the nitrogen status of the canopy. These measurements can be used to assess N uptake and be indicative of NUE parameters. As spectral measurements are non-destructive there is the opportunity to measure in real time, continuous kinetics of N uptake and utilization.

Further applications of these phenotyping approaches will aid pre-breeding and breeding programmes for improved varieties, improved management practices, and better understanding of environmental impacts, and will advance the development of precision farming technologies. Technology, both hardware and interpretive algorithms developed as a result of these platforms can be transferred to less sophisticated and cheaper devices suitable for mass use by growers. Together these advances in accurate and high-resolution monitoring of crop performance will facilitate crop production, best agronomic practice, and minimize environmental impacts on broad field commercial cropping.

## Author Contributions

MH and AR contributed equally to all aspects of this manuscript.

## Funding

Rothamsted Research receives support from the Biotechnology and Biological Sciences Research Council (BBSRC) of the UK, and this work was funded by the Designing Future Wheat project (BB/P016855/1), and the Department for Environment, Food and Rural Affairs (Defra) sponsored Wheat Genetic Improvement Network project (CH1090).

## Conflict of Interest

The authors declare that the research was conducted in the absence of any commercial or financial relationships that could be construed as a potential conflict of interest.
